# Immunological Profile and Predisposition to Autoimmunity in Girls With Turner Syndrome

**DOI:** 10.3389/fendo.2018.00307

**Published:** 2018-06-04

**Authors:** Aneta Monika Gawlik, Elzbieta Berdej-Szczot, Dorota Blat, Renata Klekotka, Tomasz Gawlik, Ewa Blaszczyk, Magdalena Hankus, Ewa Malecka-Tendera

**Affiliations:** ^1^Department of Paediatrics and Paediatric Endocrinology, School of Medicine in Katowice, Medical University of Silesia, Katowice, Poland; ^2^Department of Laboratory Diagnostics, Upper-Silesian Paediatric Health Centre, Katowice, Poland; ^3^Nuclear Medicine and Endocrine Oncology Department, Maria Skłodowska-Curie Memorial Institute and Cancer Centre, Gliwice Branch, Gliwice, Poland

**Keywords:** Turner syndrome, autoimmunity, lymphocytes subpopulation, T regulatory cells

## Abstract

**Objective:**

The risk of autoimmune diseases (AD) in patients with Turner Syndrome (TS) is twice higher than in the general female population and four times higher than in the male population. The causes of the increased incidence of AD in TS are still under discussion. We hypothesized the presence of a specific humoral, cellular, and regulatory T cell (Treg) immunity profile which predisposes to AD, disorders of immunity, and disorders of immune regulation.

**Methods:**

The study encompassed 37 girls with TS and with no signs of infection. The control group included 11 healthy girls with no hormonal disorders. A medical history focused on AD and immunity disorders was taken from all participants. The levels of: immunoglobulins IgG, IgA, IgM, total lymphocytes, lymphocytes subpopulations CD3+, CD4+, CD8+, CD19+, natural killer cells, Treg cells (CD4+ CD25+ CD127− FOXP3+), anti-inflammatory cytokines (interleukin-10, transforming growth factor-β), anti-nuclear antibodies, glutamic acid decarboxylase (GAD65 Abs), anti-thyroid peroxidase (anti-TPO Ab), and anti-thyroglobulin (anti-TG Ab) autoantibodies were determined in each participant.

**Results:**

The mean age and BMI in the TS group and in controls were comparable (11.9 ± 4.1 vs. 12.5 ± 4.0 years; 19.2 ± 3.4 vs. 19.7 ± 4.6, *p* > 0.05). Mean hSDS was significantly higher in controls (−2.2 ± 0.9 vs. −0.4 ± 1.5, *p* < 0.0001). AD and recurrent otitis media with complications were previously confirmed in 9 (24.3%) and 10 (27.0%) girls with TS. The TS group had significantly lower levels of IgG (*p* = 0.02), lower%CD4 (*p* < 0.001) and a significantly lower CD4:CD8 ratio than the controls (*p* < 0.001). There were no differences in mean Treg% between girls with TS and healthy controls. However, comparing Treg% between the TS group with coexisting autoimmunity and the remaining participants, a statistically significant difference was observed (2.09 ± 0.5 vs. 2.77 ± 1.6, *p* = 0.048). Patients with iXq had lower CD4% and more frequently had positive anti-TPO Ab and anti-TG Ab compared to the remaining girls with TS and controls (*p* = 0.001, *p* < 0.001, *p* = 0.01).

**Conclusion:**

TS predisposes to AD, especially if associated with coexisting iXq. Our preliminary findings show that patients with TS may present a specific profile of humoral and cellular immunity markers, different from healthy girls.

## Introduction

With an incidence of 1 in 2,000–2,500 liveborn female infants, Turner Syndrome (TS) is one of the most frequent chromosomal aberrations. It is characterized by short stature, incorrect gonadal development with puberty disorders, kidney, and circulatory system diseases, recurrent otitis, impaired hearing and an increased incidence of autoimmune diseases (AD).

The risk of ADs in patients with TS is approximately twice as high as in the general female population and four times higher than in males. The most common autoimmune disorder associated with TS is Hashimoto’s thyroiditis; other disorders include: celiac disease, type 1 diabetes mellitus, vitiligo, alopecia areata, ulcerative colitis, Crohn’s disease, psoriasis, idiopathic thrombocytopenic purpura, and juvenile rheumatoid arthritis (1–3). The pathogenesis of AD is complex and depends on numerous mechanisms, such as family factors connected with the HLA haplotype, genetic factors related to single nucleotide polymorphism or cytotoxic T-lymphocyte-associated protein-4 (CTLA-4), present for example on regulatory T cells (Tregs). One of the mechanisms leading to disorders of immune regulation is lack of Tregs-dependent immunosuppression (4).

Regulatory T cells are specialized T cells with the phenotype CD4+ CD25+ FoxP3+, whose function is to maintain self-tolerance and immune homeostasis by suppressing the activation, proliferation, and effector functions of various immune cells. Tregs mediate suppressive function through a variety of mechanisms and functional specialization depending on the type and location of the immune response. Tregs express CD4 and CD25 surface antigens, and intracellular forkhead family transcription factor (FoxP3)—crucial for their suppressor function. FoxP3 is coded by *AIRE*, which is located on the X-chromosome. Mutation in this gene results in disorders of tolerance to organ-specific antigens. One function of Tregs involves the secretion of interleukin-10 (IL-10), which inhibits effector T cells responses. Tregs also secrete transforming growth factor-β (TGF-β) to induce conventional T cell by FoxP3 to differentiate into Treg. As Tregs control the peripheral immune response, their potential role in the development of AD has been hypothesized.

The increased risk of autoimmunity in patients with TS has also been attributed to X-chromosome haploinsufficiency, maternal origin of the X-chromosome, excessive production of proinflammatory cytokines (IL-6), decrease in anti-inflammatory cytokines (IL-10, TGF-β), or hypogonadism (5, 6).

The impact of three copies of genetic material on the long arm of the X-chromosome and an increased incidence of AD in girls with the iXq karyotype have also been suggested (7, 8). Despite the importance of early detection and treatment of AD, literature reports are ambiguous, and studies related to girls with TS are very few. Accordingly, the aim of our study was to analyze the importance of markers of humoral, cellular, and Treg lymphocyte immunity profiles in girls with TS in determining their predisposition to AD or disorders of immunity/immunoregulation. Additionally, the impact of karyotype on autoimmunization in TS was assessed.

## Patients and Methods

The study included 37 unselected, consecutive girls with TS treated at the Department of Pediatric Endocrinology, and 11 healthy controls. The immunological profile and the presence of clinical or preclinical autoimmune disorder markers were assessed.

All the girls with TS were under treatment with recombinant growth hormone (GH, 47–66 μg/kg/day); the control group was composed of healthy girls with no hormone-related disorders. A detailed history of chronic diseases was taken from all study participants. The exclusion criteria were: an ongoing inflammatory process or lack of informed consent to participate in the study.

### Laboratory Parameters

Venous blood samples were drawn from the antecubital vein in the morning, after overnight fasting. Immunoglobulins IgG, IgA, IgM, total lymphocytes, lymphocytes subpopulations CD3+, CD4+, CD8+, CD19+, natural killer (NK) cells, Treg cells (CD4+ CD25+ CD127− FOXP3+), anti-inflammatory cytokines (IL-10, TGF-β), anti-nuclear antibodies (ANA), glutamic acid decarboxylase (GAD65 Abs), anti-thyroid peroxidase (anti-TPO Ab), and anti-thyroglobulin (anti-TG Ab) autoantibodies were analyzed. Additionally, insulin-like growth factor 1 (IGF1) was assessed in TS patients.

The numbers and percentages of lymphocyte subpopulations were determined using a standardized 4- color FACS-analysis on Becton-Dickinson cytometer (BD FAX-Caliber) and commercial reagents. CD19+ was marker for B-cells and CD3+ for T-cells, CD3+ CD4+ for helper T-cells, CD3+ CD8+ for cytotoxic T-cells, CD56+ CD3− for NK-cells. The ratio of CD4+/CD8+ was also calculated. The Human Treg cocktail (BD Pharmingen™), a three-color reagent, was used to identify the natural Treg cell population. The expression pattern of CD4+ CD25*int*/brightCD27*dim* correlated with the expression of the transcription factor Forkhead box P3 (FoxP3), a specific marker of Tregs.

Transforming Growth Factor-β was analyzed using a solid-phase enzyme-linked immunosorbent assay based on the sandwich principle (DRG TGF-β ELISA Kit). Human interleukin -10 (IL-10) was analyzed by immunoenzymetric assay, a solid-phase Enzyme Amplified Sensitivity Immunoassay performed on microtiterplate (DIAsource IL-10-EASIA). Enzyme immunoassay (Medizym anti-GAD) was used to determine GAD65 Abs in human serum. Anti-TPO Ab and anti-TG Ab concentrations were determined with radioimmunoassay (Izotop, Hungary). Values above the manufacturer-defined assay cutoff points were considered positive. IGF1 was measured by solid-phase enzyme-labeled chemiluminescent immunometric assays (IMMULITE, DPC).

The results were compared to published age-related in-house reference ranges, except for Treg, IL-10, and TGF-β, which were compared between TS and control group.

### Analysis of Karyotype Impact

In order to determine the impact of the presence of isochromosomes for the long arm of the X-chromosome (iXq) in the karyotype of blood lymphocytes on the presence of autoimmunity disorders, a subgroup of girls with iXq was identified and compared with the remaining girls with TS (TS non-iXq) and controls.

### Statistical Analysis

Statistical analyses were performed with STATISTICA version 13. Comparisons between two groups were performed with two-sided Student’s *t*-test or Student’s *t*-test with separate variance estimates, as appropriate. Comparisons between three groups were performed using ANOVA or Kruskal–Wallis rank ANOVA, as appropriate. Linear correlation analyses were used to asses IGF1 influence. Data are presented as means and SDs, and percentages. *P*-Values of <0.05 were considered significant.

The study was conducted in accordance with the Declaration of Helsinki and was approved by the Ethical Committee of the Medical University of Silesia. Written informed consent was obtained from the participants’ parents or legal custodians and from all participants aged over 16.

## Results

The study encompassed 48 girls aged from 3.4 to 18.2 years old. The median (range) age was 12.8 years (min. 3.4, max. 18.2) in the TS group and 12.8 years (min. 6.3, max. 17.9) in the control group. The detailed characteristics of the study group and controls are presented in Table 1. Out of the 37 girls with TS (study group), 9 (24.3%) had a previously confirmed AD: Hashimoto’s disease (7), psoriasis (1), celiac disease (1), and vitiligo (1); two girls had coexisting thyroiditis and celiac disease or vitiligo. Except for one girl with coexisting Hashimoto’s diseases and celiac disease (both diagnosed 6 years earlier), all the remaining girls with a confirmed AD had high levels of anti-thyroid antibodies.

**Table 1:** Clinical parameters—girls with TS vs. healthy controls (mean ± SD).

	TS (*n* = 37) mean ± SD	CG (*n* = 11) mean ± SD	*p*-Value
Age (years)	11.9 ± 4.1	12.5 ± 4.0	NS
Weight (kg)	36.7 ± 14.8	45.2 ± 19.3	NS
Height (cm)	134.8 ± 20.0	146.3 ± 21.3	NS
hSDS	−2.16 ± 0.95	−0.41 ± 1.5	<0.0001
BMI	19.2 ± 3.3	19.7 ± 4.6	NS

*TS, Turner syndrome; CG, control group; n, number of patients; BMI, body mass index; hSDS, height standard deviation score; NS, not significant*.

Positive anti-TPO Ab and/or anti-TG, together with normal thyroid function (clinical and laboratory assessment) were observed in five girls in the study group. The controls showed no signs of an AD and their antibodies test was negative. No ANA and anti-GAD were observed in any of the girls. Recurrent otitis media with complications was observed in 10 (27.0%) girls in the study group and in none of the controls.

All the study participants had a normal leukocyte count (≥4.0 × 10^3^/μl), and none had neutropenia. Six (16.2%) girls from the study group and one (9.1%) control had a slightly lowered lymphocyte count, ranging from 1.5 × 10^3^/μl to 4.6 × 10^3^/μl, but not below 1 × 10^3^/μl.

The mean leukocyte and lymphocyte counts did not differ significantly (*p* > 0.05) between the study group and controls: 6.68 × 10^3^ and 2.23 × 10^3^/μl vs. 7.25 × 10^3^ and 2.03 × 10^3^/μl.

Severe immune deficiency was excluded in all the study participants based on the analysis of concentrations of all immunoglobulin classes, and the percentages and absolute counts of lymphocyte subpopulations. The comparison of mean immunoglobulin levels showed significantly lower levels of IgG in the study group than in controls; no difference was observed for the remaining immunoglobulin classes (Table 2). None of the immunoglobulin levels were lower than normal.

**Table 2:** Laboratory markers of humoral and cellular immunity, and anti-inflammatory cytokines in girls with TS vs. healthy controls (mean ± SD).

	TS (*n* = 37) mean ± SD	CG (*n* = 11) mean ± SD	*p* Value
IgA (g/l)	1.08 ± 0.39	1.15 ± 0.42	NS
IgM (g/l)	0.84 ± 0.30	1.01 ± 0.42	NS
IgG (g/l)	9.14 ± 2.10	11.15 ± 3.29	0.019
TGF-β (pg/ml)	260.2 ± 121.9	195.2 ± 113.7	NS
IL-10 (pg/ml)	7.38 ± 2.30	19.23 ± 38.39	NS
CD3 (%)	66.9 ± 7.3	68.2 ± 6.0	NS
CD3 (cells/μl)	1369.5 ± 504.8	1387.8 ± 531.4	NS
CD4 (%)	32.6 ± 5.5	40.4 ± 5.8	NS
CD4 (cells/μl)	656.3 ± 227.3	817.3 ± 350.1	<0.001
CD8 (%)	28.6 ± 6.4	24.3 ± 5.4	NS
CD8 (cells/μl)	597.6 ± 272.6	494.8 ± 188.3	NS
CD19 (%)	16.4 ± 4.4	15.7 ± 4.9	NS
CD19 (cells/μl)	326.0 ± 108.9	329.4 ± 189.7	NS
NK (%)	14.3 ± 6.6	13.9 ± 6.5	NS
NK (cells/μl)	290.9 ± 171.2	263.8 ± 132.7	NS
Treg (%)	2.76 ± 1.56	2.37 ± 1.18	NS

*TS, Turner syndrome; CG, control group; *n*, number of patients; NS, not significant; IgA, IgM, IgG, immunoglobulins IgA, IgM, IgG; CD3, CD4, CD8, CD19, lymphocytes subpopulations CD3+, CD4+, CD8+, CD19+; NK, natural killer cells, Treg cells, regulatory T cells (CD4+ CD25+ CD127− FOXP3+); IL-10, interleukin-10; TGF-β, transforming growth factor-β*.

With regard to lymphocyte subpopulation, the level of CD4+ lymphocytes was significantly lower in girls with TS than in controls (Table 2). There were no significant deficiencies in lymphocyte subpopulations. The levels of absolute counts of lymphocytes CD3+, CD4+, CD8+, and CD19+ were slightly lower than normal for age in 4 (10.8%) girls with TS and in 1 (2.7%) control.

The study group and the controls differed significantly with regard to the CD4:CD8 ratio (1.20 vs. 1.77, *p* < 0.001). In 10 girls, the CD8+ cell count was higher than the CD4+, or equal: 9 (24.3%) girls with TS and 1 (9.1%) control. In five (13.5%) girls with TS, an abnormal CD4:CD8 ratio was observed; four of the girls presented clinical and/or laboratory signs of AD.

The percentage of Treg cells (Treg%) was between 1.2 and 7.42% in the study group and between 1.2 and 4.9% in the control group. The mean values of IL-10 and TGF-β concentrations did not differ between the study group and the controls (*p* > 0.05) (Table 2). The mean values of Treg% in girls with TS and coexisting autoimmunity were significantly lower than Treg% in the remaining participants (2.09 ± 0.5 vs. 2.77 ± 1.6, *p* = 0.048). No correlation was observed between Treg% and the levels of cytokines IL-10 and TGF-β.

Weak correlations between IGF1 concentrations (in reference ranges) and some markers of the immune profile in TS were found: negative with CD3+, CD8+, CD19+, NK cell counts (*r*^2^ = 0.22, *p* = 0.016; *r*^2^ = 0.17, *p* = 0.04; *r*^2^ = 0.23, *p* = 0.014; *r*^2^ = 0.25, *p* = 0.009, respectively) and positive with anti-TG Ab (*r*^2^ = 0.17, *p* = 0.015).

Seven girls with iXq were identified in the study group. They presented significantly lower CD4% and more frequently had elevated anti-TPO Ab and anti-TG Ab antibody titers compared to the remaining girls with TS and controls (*p* = 0.001, *p* < 0.001, *p* = 0.01). Girls with three copies of genes from Xq also presented the lowest Treg and the highest CD8, though the levels were not statistically significant (Table 3).

**Table 3:** Laboratory markers of humoral and cellular immunity, and anti-inflammatory cytokines in girls with TS and the iXq karyotype vs. girls with TS non-iXq and vs. healthy controls (mean ± SD).

	TS iXq (*n* = 7) mean ± SD	TS non-iXq (*n* = 30) mean ± SD	CG (*n* = 11) mean ± SD	*p* Value
IgA (g/l)	0.92 ± 0.43	1.11 ± 0.37	1.15 ± 0.42	NS
IgM (g/l)	0.87 ± 0.32	0.83 ± 0.31	1.01 ± 0.42	NS
IgG (g/l)	9.27 ± 3.07	9.11 ± 1.88	11.15 ± 3.29	NS
TGF-β (pg/ml)	209.8 ± 93.6	271.9 ± 126.0	195.2 ± 113.7	NS
IL-10 (pg/ml)	7.70 ± 2.09	7.30 ± 2.38	19.23 ± 38.39	NS
CD3 (%)	67.2 ± 8.1	66.8 ± 7.2	68.2 ± 6.0	NS
CD3 (cells/μl)	1374.3 ± 613.6	1368.2 ± 488.6	1387.8 ± 531.4	NS
CD4 (%)	30.8 ± 5.8	33.1 ± 5.5	40.4 ± 5.8	NS
CD4 (cells/μl)	625.7 ± 287.6	664.3 ± 216.0	817.3 ± 350.1	0.001
CD8 (%)	30.2 ± 6.5	28.2 ± 6.4	24.3 ± 5.4	NS
CD8 (cells/μl)	620.0 ± 294.1	591.8 ± 273.4	494.8 ± 188.3	NS
CD19 (%)	14.2 ± 2.0	16.9 ± 4.7	15.7 ± 4.9	NS
CD19 (cells/μl)	279.3 ± 102.0	338.1 ± 109.4	329.4 ± 189.7	NS
NK (%)	16.2 ± 7.3	13.8 ± 6.5	13.9 ± 6.5	NS
NK (cells/μl)	295.2 ± 112.8	289.7 ± 185.5	263.8 ± 132.7	NS
Treg (%)	1.84 ± 0.40	2.99 ± 1.66	2.37 ± 1.18	NS

*TS, Turner syndrome; iXq, isochromosome Xq; CG, control group; *n*, number of patients; NS, not significant; IgA, IgM. IgG, immunoglobulins IgA, IgM, IgG; CD3, CD4, CD8, CD19, lymphocytes subpopulations CD3+, CD4+, CD8+, CD19+; NK, natural killer cells, Treg cells, regulatory T cells (CD4+ CD25+ CD127− FOXP3+); IL-10, interleukin-10; TGF-β, transforming growth factor-β*.

## Discussion

Every fourth girl with TS in our study group was diagnosed with an AD. This is in line with our previous findings and literature data, according to which approx. 20–50% of patients with TS, depending on their age, are diagnosed with an AD (1, 8–13). Chronic thyroiditis (Hashimoto’s disease) was confirmed in almost a fifth of the patients; few also suffered from psoriasis, celiac disease, or vitiligo (the last two were associated with thyroiditis).

In compliance with the protocol, test exclusion criteria were: an acute infection, a recent acute disease, or a recent vaccination. Recurrent otitis, frequently with complications, was noted in the history of every fourth girl with TS. However, in only two of them, a slight deviation in immunological tests was noticed, though it was not significant from a clinical point of view. We did not observe significant immunodeficiency regarding the levels of immunoglobulins or the main lymphocyte subpopulations, which is in line with the Karolinska University Hospital reports from 2004 (14). As observed in the early 80s by Cacciari et al., also in our study, girls with TS had relatively lower IgG levels than the controls (15).

Stenberg’s analyses of patients with an increased risk of disorders of immune tolerance revealed a low percentage of CD4 and a lower CD4:CD8 ratio, just as in our study group (14). A low CD4:CD8 ratio in the population of patients with TS has also been confirmed by Maureen et al. (16).

One of the mechanisms of immune regulation disorders may be the loss of control by Tregs as a result of their absence or dysfunction. Therapeutic administration of Tregs (experimental stage) with a view to stop the development of AD, e.g., type 1 diabetes, is being tested (17). We determined the percentages of CD4+ CD25+ CD127− FoxP3+ T cells phenotype and the results obtained in our study were lower than those reported in the literature (18). Like Maureen et al. (16), we observed no differences in Treg% between girls with TS and the controls. However, the percentage of Tregs in girls with TS and autoimmunity was significantly lower than in the remaining study participants.

Similar results were obtained in two studies on juvenile idiopathic arthritis (JIA) (19, 20). In Wei’s study, a lower T phenotype CD4+ CD25^high^ lymphocyte count, with simultaneous lower expression of CTLA-4 was confirmed in a population with JIA. The opposite was observed by Sznurkowska et al. (21), whereby the percentage of Tregs was higher in children with JIA than in controls; however, the tests were performed in newly diagnosed children before treatment.

There are contradictory results concerning Tregs depending on the selected study group (19–21). It is highly probable that the results may be influenced by the length of the autoimmunity process, degree of compensation, and organ damage.

Similar to our analysis, also other studies with a control group (21) did not confirm the presence of a relationship between the autoimmunity process and anti-inflammatory cytokine secretion (IL-10 and TGF-β). Additionally, in our study we did not observe any differences in the levels of these cytokines between girls with TS and coexisting AD, and girls with TS without coexisting AD.

In the subgroup of girls with isochromosome Xq, we observed a significantly higher frequency of autoimmunity, particularly regarding the thyroid. Similar results were obtained by other authors (7, 8). The Oxford centre (7) confirmed that in a group of 145 women with TS, over 80% of those with iXq had positive anti-thyroid antibodies. In an Italian study (8), nearly 40% of a group of 66 patients with TS had thyroiditis, significantly more frequently in the subgroup with the iXq karyotype.

We are aware of the limitations of our study. Our results may be biased by the relatively small number of participants, especially in the control group. The power analysis showed that 25 controls were needed for our TS group of 37 patients in order to have a strong effect size with Cohen’s *d* of 0.8 in the *t*-test. Unfortunately, we were only able to recruit 11 controls, which gives the power of 0.63. The study was conducted in a population of children and only age-matched healthy girls were involved. The small number of girls with TS could be explained by the incidence of TS in the general population and the prospective nature of the study. Future work should also consider comparing laboratory autoimmune markers between TS patients and non-TS population with autoimmune disorders. Patients with confirmed hypogonadism or hypothyroidism were under hormone therapy. Most of our girls with TS were treated with GH or had already completed the treatment. The GH receptor expression on immune cells (on more than 90% of B lymphocytes and monocytes, but only variably on T lymphocytes and NK cells) could suggest the presence of an impact of GH on the immune system, though it has not been fully explored yet (22–24). The low GH receptor number expressed on peripheral blood lymphocytes was confirmed by Bresson et al. (25). Studies on the effect of GH therapy both in GH deficient and non-GH deficient children on immune functions have given discrepant results; however, in most of them without significant changes (26–30). Similarly, little attention has been given to the interaction between GH and cytokines, and the published results seem to be ambiguous, or even contradictory (31). At this point, it is difficult to give a definite answer as to whether the therapy used in our patients had any impact on the obtained results. Our analysis showed only weak correlation between IGF1 and some of the immunological parameters: higher normal IGF1 concentrations corresponded with lower counts of CD3+, CD8+, CD19+, NK cells and higher anti-TG Ab.

Our results confirm a higher incidence of AD in the population with TS, especially with predisposition to autoimmunity in patients with iXq. Among the laboratory markers confirming abnormalities of humoral and cellular immunity, our attention was drawn to the low levels of immunoglobulin G, low percentage of Tregs and the low CD4:CD8 ratio. However, in view of the study limitations, our results should be considered preliminary.

The latest guidelines emphasize that the risk of AD increases with age; therefore, regular follow-up and screening are recommended, both in children and adults (e.g., thyroid function at diagnosis and thereafter annually; celiac screen starting at the age of 2, and thereafter every 2 years) (32). Identifying a specific immunological profile in patients with TS and autoimmune disease(s) could potentially be relevant in everyday clinical practice.

At present, AD are mainly treated with supplementation, in the case of organ-specific diseases, or by inflammation suppression, in the case of systemic diseases. Broadening the knowledge about disorders of immune regulation and loss of immune tolerance, especially with regard to Tregs activation, may provide new methods of therapy, both for the prevention and suppression of autoimmune disorders.

## Ethics Statement

The study was conducted in accordance with the Declaration of Helsinki and was approved by the Ethical Committee of the Medical University of Silesia. Written informed consent was obtained from the participants’ parents or legal custodians and from all participants aged over 16.

## Author Contributions

AG and EB-S designed the study, analyzed the database, and wrote the manuscript, their contribution was equal. TG, EB, MH, and EM-T prepared and analyzed the patient database and wrote the manuscript. DB and RK collaborated in designing the work and performed laboratory analyses.

## Conflict of Interest Statement

The authors declare that the research was conducted in the absence of any commercial or financial relationships that could be construed as a potential conflict of interest.
